# Supplementary material for: Mechanisms governing the pioneering and redistribution capabilities of the non-classical pioneer PU.1
Source: Nat Commun. 2020 Jan 21;11:402. doi: 10.1038/s41467-019-13960-2 (PMC6972792; doi:10.1038/s41467-019-13960-2)
Supplement: Supplementary file 7 — Source data [file 41467_2019_13960_MOESM7_ESM.zip › Source_Data/FigureS5/FigureS5B_MotifsETS1specificPeaks/homerResults/motif1.similar.html]

motif1

## Information for motif1

G
T
C
A
G
T
A
C
A
G
C
T
G
T
C
A
A
G
T
C
T
C
G
A
C
T
G
A
G
A
C
T
A
G
C
T
G
A
T
C
A
G
T
C
G
T
A
C
C
T
G
A
A
C
T
G
  
Reverse Opposite:  

G
T
A
C
G
A
C
T
C
A
T
G
C
T
A
G
C
A
T
G
T
C
G
A
C
T
G
A
G
A
C
T
A
G
C
T
T
A
C
G
C
A
G
T
C
T
G
A
A
C
T
G
C
A
G
T
  

|  |  |
| --- | --- |
| p-value: | 1e-205 |
| log p-value: | -4.723e+02 |
| Information Content per bp: | 1.639 |
| Number of Target Sequences with motif | 355.0 |
| Percentage of Target Sequences with motif | 31.28% |
| Number of Background Sequences with motif | 188.7 |
| Percentage of Background Sequences with motif | 2.31% |
| Average Position of motif in Targets | 101.8 +/- 44.6bp |
| Average Position of motif in Background | 102.4 +/- 78.3bp |
| Strand Bias (log2 ratio + to - strand density) | 0.2 |
| Multiplicity (# of sites on avg that occur together) | 1.50 |
| Motif File: | file (matrix) reverse opposite |

### Similar de novo motifs found

|  |  |  |  |  |  |  |  |
| --- | --- | --- | --- | --- | --- | --- | --- |
| Rank | Match Score | Redundant Motif | P-value | log P-value | % of Targets | % of Background | Motif file |
| 1 | 0.983 | T G C A G T A C A G C T T C G A A G T C T C G A C G T A A G T C G A C T G A T C A G T C G T A C C T G A | 1e-199 | -459.223901 | 31.37% | 2.50% | motif file (matrix) |
| 2 | 0.956 | G T C A G A T C A G C T T G C A G A T C T C G A G C T A A G C T G A C T G A T C A G T C G T A C | 1e-191 | -440.711142 | 31.01% | 2.65% | motif file (matrix) |
| 3 | 0.820 | C T A G C T A G T G C A A G T C A C G T G T C A A G T C T G C A G C T A G A T C G A C T G A T C A G T C | 1e-173 | -400.047751 | 30.31% | 3.03% | motif file (matrix) |
| 4 | 0.900 | G A T C A G C T C G T A A T G C C T G A C T G A G A C T G A C T G A T C A G T C G T A C | 1e-170 | -392.673593 | 30.57% | 3.24% | motif file (matrix) |
| 5 | 0.767 | C G T A A G T C A C G T C G T A A T G C C T G A C G T A C G T A G A C T | 1e-127 | -294.308040 | 27.40% | 3.90% | motif file (matrix) |
| 6 | 0.836 | G A T C A G C T C T G A G T A C T C G A A T C G A G T C G C A T A T G C A G T C G T A C C T G A | 1e-116 | -269.205494 | 22.47% | 2.57% | motif file (matrix) |
| 7 | 0.801 | A C T G A C T G C A G T G C T A A C G T A G C T A G T C A C T G G A C T C G T A A T C G A C G T A G C T A G C T | 1e-62 | -143.646050 | 8.63% | 0.40% | motif file (matrix) |
